# Bridging Policy and Service Performance of Hospital-Based Nutrition Support by Healthcare Information Technology

**DOI:** 10.3390/nu13020595

**Published:** 2021-02-11

**Authors:** Jungwon Cho, Young Suk Park, Do Joong Park, Soyeon Kim, Haekyung Lee, Minjeong Kim, Eunsook Lee, Ho-Young Lee, Euni Lee

**Affiliations:** 1 Department of Pharmacy, Seoul National University Bundang Hospital, Gumi-ro 173, Bundang-gu, Seongnam-si, Gyeonggi-do 13620, Korea; xcully@snubh.org (J.C.); escduck@snubh.org (E.L.); 2 College of Pharmacy & Research Institute of Pharmaceutical Sciences, Seoul National University, Gwanak-ro 1, Gwanak-gu, Seoul 08826, Korea; 3 Department of Surgery, Seoul National University Bundang Hospital, Gumi-ro 173, Bundang-gu, Seongnam-si, Gyeonggi-do 13620, Korea; youngsukmd@gmail.com; 4 Department of Surgery, Seoul National University Hospital, Daehak-ro 101, Jongno-gu, Seoul 03080, Korea; dojoongpark@gmail.com; 5 Department of Surgery, Seoul National University College of Medicine, Daehak-ro 101, Jongno-gu, Seoul 03080, Korea; 6 Department of Clinical Nutrition, Seoul National University Bundang Hospital, Gumi-ro 173, Bundang-gu, Seongnam-si, Gyeonggi-do 13620, Korea; juniffer@snubh.org; 7 Department of Nursing, Seoul National University Bundang Hospital, Gumi-ro 173, Bundang-gu, Seongnam-si, Gyeonggi-do 13620, Korea; pipo7474@snubh.org; 8 Office of Quality Improvement and Process Innovation, Seoul National University Bundang Hospital, Gumi-ro 173, Bundang-gu, Seongnam-si, Gyeonggi-do 13620, Korea; phebe7@snubh.org; 9 Office of Digital Medicine, Seoul National University Bundang Hospital, Gumi-ro 173, Bundang-gu, Seongnam-si, Gyeonggi-do 13620, Korea; 10 Department of Nuclear Medicine, Seoul National University Bundang Hospital, Seoul National University College of Medicine, Gumi-ro 173, Bundang-gu, Seongnam-si, Gyeonggi-do 13620, Korea

**Keywords:** health information system, nutrition support practice, nutrition support team, service quality improvement

## Abstract

Although the healthcare policy was implemented to incentivize the multidisciplinary services of hospital-based nutrition support team (NST) in South Korea, timely completion of the services has been challenging in the hospitals. We enhanced NST healthcare information technology (NST−HIT) to bridge the gap between policy implementation and seamless execution of the policy in the hospital system. A 48 month pre-test−post-test study was performed, including a 12 month pre-intervention period, a six month intervention period, and a 30 month post-intervention period. The enhanced NST−HIT provided sufficient patient data and streamlined communication processes among end-users. A Student’s *t*-test showed that the timely completion rate of NST consultations, the reimbursement rate of NST consultations, average response times of NST physicians and nurses, and length of hospital stay significantly improved during the post-intervention period. A segmented regression analysis of interrupted time series showed that the average response times of NST physicians had sustained after the interventions. We believe that well-structured, multi-pronged initiatives with leadership support from the hospital improved service performance of hospital NST in response to national-level healthcare policy changes.

## 1. Introduction

Nutrition support is a significant factor closely related to clinical outcomes in hospitalized patients receiving various therapies [1,2,3]. Previous studies have indicated that improved nutritional status is associated with a reduced length of hospital stay [4,5] and unplanned readmission rates [4,6]. A multidisciplinary approach by the nutrition support team (NST) is introduced to screen patients’ nutritional status and support appropriate nutritional therapy in hospitals [7,8]. The preemptive role of NSTs is emphasized in hospitals based on previous studies, in that nutritional support is associated with improved survival and nonelective hospital readmission rates during hospitalization [9].

In August 2014, the Korean Ministry of Health and Welfare announced a new policy change of health insurance on hospital-based nutrition services called “*Therapy by Nutrition Support Teams*”. With the policy change, multidisciplinary NSTs were established [10]. Reimbursement for the NST services was assured by the payer if the following requirements were met: (1) participating hospitals must have multidisciplinary NSTs, consisting of attending physicians, pharmacists, nurses, and dietitians; (2) all NST members must be certified by the Korean Society for Parenteral and Enteral Nutrition and registered with the Healthcare Insurance Review and Assessment Service (HIRA); (3) a designated member should serve as the NST manager for handling and processing reimbursement claims.

**Figure 1:**
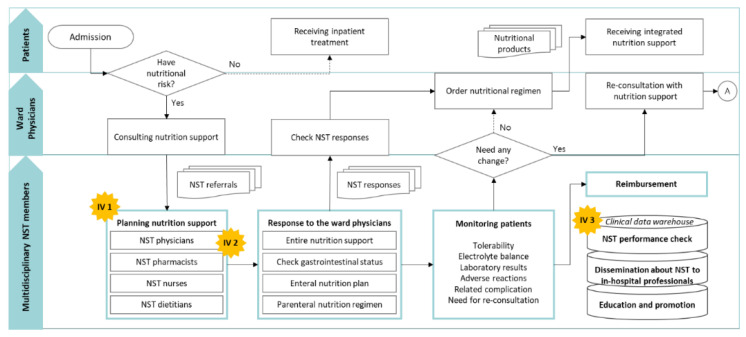
Nutrition support process by the multidisciplinary nutrition support team (NST) at Seoul National University Bundang Hospital: Intervention (**IV 1**), NST patient management program establishment; (**IV 2**) upgrade of NST attending physicians’ response steps; (**IV 3**) construction of clinical data warehouse.

NST physicians are responsible for entire nutrition support plans and coordinated care, overseeing the status of nutritional supports for patients based on gastrointestinal symptoms, imaging studies, and diagnosis. NST nurses record patients’ gastrointestinal symptoms and nutritional status—residual amount, nausea, vomiting, aspiration tendency, bowel sound, defecation count, and blood glucose control—during patient care and observation. NST dietitians are in charge of designing enteral nutrition and oral diet; they interview patients in admission, perform nutritional assessments, calculate nutrition requirements, and plan optimal enteral formulas. NST pharmacists develop parenteral nutritional plans based on weight, disease, laboratory results, drug−nutrient interactions, and adverse reactions. The process of nutrition support by the multidisciplinary NST at Seoul National University Bundang Hospital (SNUBH) was arranged similarly to the literature [11] as presented in Figure 1.

The policy changed the healthcare environment in South Korea and promoted the formation of multidisciplinary NSTs at the hospital level [10]. However, an implementation gap existed on putting the new policy and practices into efficient operation in our hospital because NST referrals of ward physicians, an essential step for timely provision of nutrition services to the patients, were often missed or delayed, primarily in anticipation of the decision made by the medical residents [12]. An enhanced NST healthcare information technology (NST−HIT) was needed to achieve more efficient communication among the NST team members for timely and collaborative completion of NST consultations, as suggested in the literature [13]. To improve the performance and service quality of NST, our research team revamped the NST−HIT structure with a goal to bridge the implementation gap between knowledge and action for nutrition care in the hospital setting by reorganizing the nutrition support system with better accessible to all NST members on all healthcare information technology (HIT) components, as depicted in Figure 1. Consequently, order confirmation processes were streamlined among team members and the authorized NST manager was able to expedite the service process.

We have conducted a study to report changes in the NST process with the improved HIT components to evaluate its impact on NST performance in response to national-level healthcare policy changes and to discuss public health implications in nutrition support services for hospitalized patients.

## 2. Materials and Methods

### 2.1. Study Design and Period

**Figure 2:**
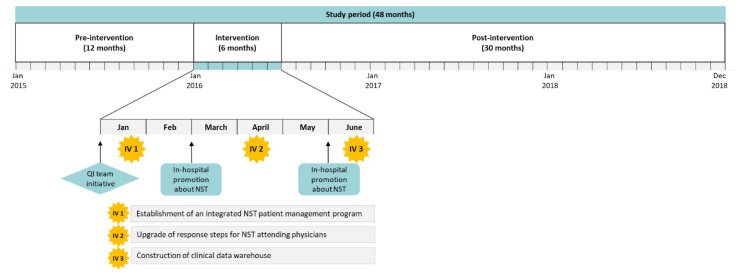
Study timeline, including 12 month pre-intervention (January 2015–December 2015) and 30 month post-intervention (July 2016–December 2018) periods. The interventions were performed within six months (January 2016–June 2016). IV, intervention; NST, nutrition support team; QI, quality improvement.

This prospective pre-test−post-test study evaluated the effect of multifaceted interventions with enhanced NST−HIT and facilitated staff engagement supported by the hospital leadership through the dissemination and periodic promotion of NST and NST−HIT changes in clinical departments. The overall study period was 48 months (January 2015–December 2018) divided into a 12 month pre-intervention (January 2015–December 2015), a six month intervention (January 2016–June 2016), and a 30 month post-intervention (July 2016–December 2018) period (Figure 2).

### 2.2. Study Site and Healthcare Information Technology

SNUBH is a 1335-bed tertiary care teaching hospital with approximately 74,000 hospitalizations annually. Since the hospital was first opened in 2003, it has been fully digitized using HIT, with electronic medical records (EMRs) of patient demographic information, progress, medications, referrals, and laboratory results. SNUBH is the first hospital outside North America certified with the highest level of HIT applications by the Healthcare Information and Management System Society [14].

For NST within the SNUBH−HIT, nutrition-related information, including the risk of malnutrition, was gathered upon the patient’s admission, and nutritional screenings were completed using a daily record of the patient’s age, body mass index, serum albumin, and total lymphocyte counts. This nutritional screening tool of SNUBH, the so-called “Nutrition Screening Index (NSI)”, was first developed based on the PG−SGA (Patient-Generated Subjective Global Assessment) in 2006 [15], and validated with NRS 2002 (Nutritional Risk Screening 2002) [16] in 2009 [17] (Table A1). According to the results of NSI, hospitalized patients were categorized into one of three malnutrition risk groups (high, low, or normal) on the first day of admission. When a patient was categorized as “malnutrition—high risk”, focused nutrition support was initiated by NST with a highlighted “NST” icon on the EMR screen. The ward physicians were then guided to make referrals to the NST for patient-specific nutritional planning.

### 2.3. Interventions

A project team was organized, including two attending physicians, a pharmacist, a nurse, and a dietitian from the NST, and a project manager, and was designated a facilitator from the office of quality improvement and process innovation in December 2015. The team mapped out the NST service and intervention plan to provide sufficient patient-related data and expedite communication among the end-users (Figure 2). Dissemination and the promotion of nutrition support to clinical departments were periodically performed.

#### 2.3.1. Enhanced Nutrition Support Practice

Multidisciplinary NSTs in SNUBH typically perform daily rounds for intensive care unit patients. Additionally, a multidisciplinary NST from the Department of Surgery began biweekly rounds with the ward physicians for inpatients who were at risk of malnutrition. For outpatients (including patients on home nutrition support), the NST commenced weekly ambulatory sessions to check patients’ nutritional statuses and design proper nutritional plans.

#### 2.3.2. Enhanced NST−HIT Components

- Component 1: Establishment of an Integrated NST Patient Management Program

**Figure 3:**
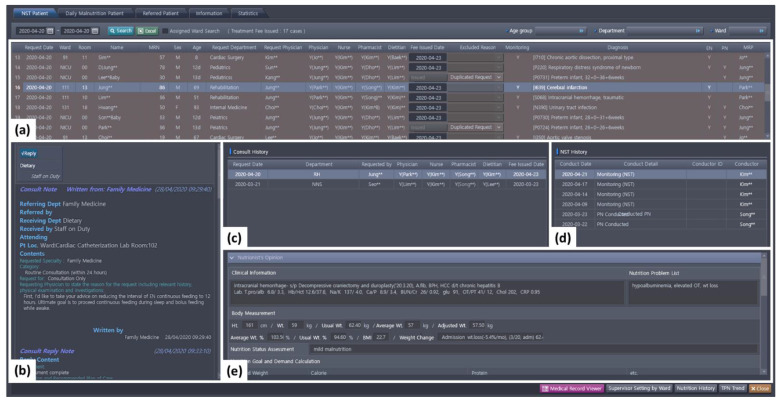
Nutrition support team (NST) patient management program at Seoul National University Bundang Hospital. The program enabled all NST members to share patient information and facilitated communication among team members. Each subsection of the screen represents the following: (**a**) list of referred patients and their information for nutrition support; (**b**) details of the referrals and responses from NST members; (**c**) NST referral history of the patient; (**d**) NST monitoring history of the patient; and (**e**) notes and opinions from NST members.

An integrated NST patient management program was newly structured to provide comprehensive real-time information, displaying patients’ nutrition-related information (e.g., demographic information, contents for the referrals, responses from the NST, and monitoring data; Figure 3). The program model aimed to maximize patient-centered nutrition support by enabling access to the screen and sharing patient information with all NST professionals. The program was designed for NST members to inquire about the entire NST referral history, and each team member representing the professional department was able to consult other members’ opinions on the program’s interface at the time of nutritional decision making.

- Component 2: Upgrade of NST Attending Physicians’ Response Steps

**Figure 4:**
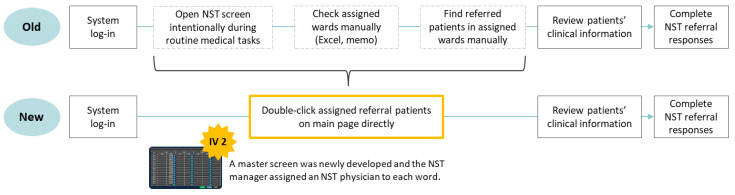
Simplified response steps by nutrition support team (NST) physicians at Seoul National University Bundang Hospital; IV2: Upgrade of NST attending physicians’ response steps.

Functions of NST attending physicians’ response steps were upgraded for easily checking and rapidly replying to enquiries regarding NST referrals. Before the upgrade, NST attending physicians performed at least six steps to complete NST referral responses, the main reason for low 48 h NST consultation completion rates (Figure 4).

A master screen was set up to allow the NST manager to assign an NST physician to each ward. If an assigned NST physician logged into the EMR, the designated NST physician could see the NST consultation requests related to the referred patients, review the patients’ clinical information in the EMR, and easily respond to the NST plan.

- Component 3: Construction of a Clinical Data Warehouse

Clinical data warehouse (CDW), a structured data repository [18,19], was built to monitor the quality of the NST process and to support research. CDW allowed NST members to check NST intervention outcomes by collecting data on NST referrals, response rates, and reimbursement rates without help from the information technology (IT) department.

#### 2.3.3. Promotion of NST to Clinical Departments

With the implementation of the enhanced nutrition support practice and NST−HIT, the team produced educational materials and made presentations at clinical meetings to promote the support system with a focus on building institutional culture and promoting multidisciplinary collaboration toward encouraging continuous quality improvement. 

The role of the ward physicians was particularly crucial to initiating nutrition support during care. Thus, we periodically furnished the ward physicians with information on the NST−HIT using promotional materials containing key facts on nutrition support for hospitalized patients. This promotion was hospital-wide and supported by leaders of clinical departments.

### 2.4. Outcomes

The primary outcome was a timely completion rate of NST consultations by all four types of professionals, including the attending physician, pharmacist, nurse, and dietitian. The completion of an NST consultation within 48 h of the issuance of an NST referral was operationally defined as the timely completion of the NST consultation. The average response time of each professional was analyzed. The secondary outcome was an NST reimbursement rate. The NST service should fulfill the reimbursement criteria by the insurance payer; therefore, the NST manager checked HIRA’s service terms and conditions daily. If the service did not meet the requirements for reimbursement in this study, the reason was recorded in the integrated NST patient management program and the data were excluded from the analysis. Consequently, the time required to order total parenteral nutrition (TPN) from the time of NST referral was recorded, to evaluate the intervention effect on actual nutrition support services for referred patients.

In addition to the above-mentioned administrative outcomes, the data on the length of hospital stays were used to evaluate the impacts of the intervention on clinical outcomes. For evaluating the length of hospital stay, the analysis included non-critically ill patients (i.e., who were hospitalized in general wards excluding intensive care units) receiving NST services. Outcome data were extracted from the CDW from January 2015 to December 2018.

### 2.5. Statistical Analysis

The timely completion rate of NST consultation, the average response time of each professional, and the NST reimbursement rate were estimated. Changes in the outcome measures were compared between the pre- and post-intervention periods using the Student’s *t*-test. A segmented regression analysis of interrupted time series [20] was used for evaluating improved response time by NST physicians. This analytic approach provided statistical estimation of changes in the level and trend between the pre- and post-intervention periods (segments). The level and trend were defined as the value at the beginning of each period (intercept) and the rate of change during each period (slope), respectively. We also performed Student’s *t*-tests for evaluating the time required to order TPN by ward physicians and length of hospital stay. Analyses were performed using R version 4.0.2 2020 (The R Foundation for Statistical Computing).

### 2.6. Ethics Approval and Consent to Participate

This study was approved by the institutional review board of Seoul National University Bundang Hospital (B−1812−510−110), and a waiver for written consent was obtained from the institutional review board.

## 3. Results

### 3.1. Changes in the Timely Completion Rate of NST Consultation

Table 1 shows mean changes in the timely completion rate of NST consultation (%) and the response time by professionals (hours) between the pre- and post-intervention periods. During the post-intervention period, the timely completion rate of NST consultation significantly improved (pre-intervention: 45.00 ± 8.24% vs. post-intervention: 82.36 ± 4.68%, *p* < 0.001).

### 3.2. Response Time by Professionals

Regarding the response time of the professionals (hours), those of NST physicians and nurses were significantly improved (Table 1). The average response time of an NST physician was significantly improved in the post-intervention period (pre-intervention: 71.77 ± 29.98 h vs. post-intervention: 19.76 ± 4.64 h, *p* < 0.001).

**Figure 5:**
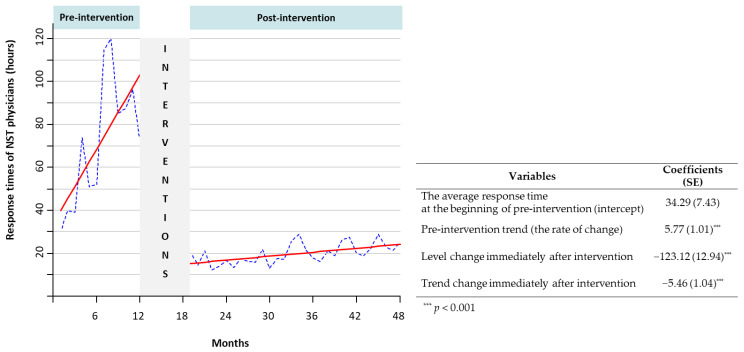
Pre- and post-intervention monthly response times (hours) of the nutrition support team (NST) physicians.

Figure 5 presents the monthly response times of NST physicians (hours) between two periods, as depicted by the dotted blue. The solid regression line in red indicates a significant monthly delay in the average response time during the pre-intervention period and a trend change closed to constant (slope changes from 5.77 h per month pre-intervention to 0.31 h per month post-intervention).

### 3.3. Changes in the NST Reimbursement Rate

The mean changes in the reimbursement rate (%) between the pre- and post-intervention periods was significantly improved (pre-intervention: 51.36 ± 9.29% vs. post-intervention: 78.65 ± 4.17%, *p* < 0.001) (Table 1).

### 3.4. Duration of TPN Order by Ward Physicians

**Figure A1:**
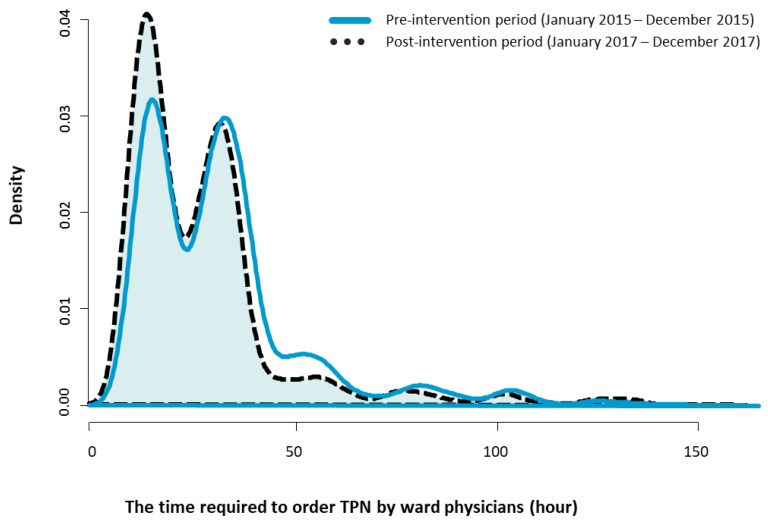
Kernel density curve of the time required to order TPN by ward physicians during pre- and post-intervention periods. TPN, total parenteral nutrition.

The average time required to order TPN by ward physicians during the pre-intervention period (January 2015–December 2015) was 22.81 ± 21.80 h, and 19.19 ± 20.38 h during the post-intervention period January 2017–December 2017 for referred patients (Table A2). With an increased timely completion rate of NST consultations, the time required to order TPN by ward physicians reduced significantly (*p* < 0.001). The kernel density curve showed the tendency of a shortened TPN order duration during the post-intervention period (Figure A1).

### 3.5. Length of Hospital Stay for Patients Receiving NST Services

Regarding the length of hospital stay as a proxy indicator of clinical outcomes, hospitalized adult patients receiving NST services in general wards were included in the analysis during pre-intervention (January 2015−December 2015, *n* = 1171) and post-intervention (January 2018–December 2018, *n* = 2171) periods (Table A3). The mean length of hospital stay was 32.87 ± 28.55 days during the pre-intervention period, and 30.42 ± 28.61 days during the post-intervention period. With the enhanced nutritional support services, our study showed that the mean length of hospital stay was significantly reduced by about two days (mean difference −2.45 days; 95% CI, −4.48, −0.41; *p* < 0.05).

## 4. Discussion

The significance of our findings is two-fold: the timely completion rate of NST consultation and reimbursement rate were significantly increased during the study periods; we have also provided evidence that hospital-wide supports and improved HIT systems bridged the gap between the newly implemented healthcare policy and successful execution of multidisciplinary NST services in South Korea, based on a four-year intervention study. We believe that more efficient NST services delivered to the hospitalized patients can serve as a proxy indicator of improved clinical performance with public health implications. 

According to Langley and Beasley, HIT potentially enables better patient care and helps clinicians achieve continuous improvements in the quality of primary healthcare [21]. The time required by ward physicians to order TPN was significantly shortened after enhanced HIT and promotion of the system. The improved NST provision was more efficient and meaningful, as a well-structured process that can provide quality services. We also assessed the length of hospital stay as a clinical outcome and an indicator of NST-related care quality. An enhanced NST service was associated with reduced lengths of hospital stays for hospitalized patients at nutritional risk. Our findings reflected that the enhanced NST service performance using NST−HIT could improve not only administrative efficiencies in nutrition care processes, but also clinical outcomes during hospitalization. Before the intervention, NST members could only check patient referral lists on their individual professional HIT system, but throughout the intervention period, they could share clinical information and perform patient-centered nutrition support via the integrated NST patient management system. Consequently, the time saved from HIT enhancement would allow healthcare professionals to increase the direct care of patients and potentially enhance patients’ long-term clinical consequences.

We believe that managing healthcare manpower issue is directly relevant to improved public health. West et al. suggested that physician burnout is prevalent due to issues with patient care, the healthcare system, and physician health [22]. According to the Organization for Economic Cooperation and Development (OECD) Health Statistics, 2018 [23], nursing staff are also scarce. Considering that the clinical environment of South Korea is highly dependent on tertiary care hospitals, any methods that would reduce the workload of health professionals and use their efforts effectively can be highly strategic. The role of IT in healthcare systems should be actively entertained in a system with healthcare manpower shortages such as South Korea. A well-integrated HIT [24,25] is imperative to Korean healthcare professionals for the efficient retrieval and recording of patients’ medical history, and effective provision of nutrition services by leveraging the manpower to embrace growing healthcare needs from the public health perspectives.

An effective HIT that was developed in our study corroborated results from other studies, regarding the improvements in efficiency, productivity, and quality of care [26,27]. Efficient HIT could help healthcare professionals access patient records more effectively, thereby increasing productivity. Although NST physicians play an important role in adequate nutrition support, they were typically unable to spend much time on NST responses due to busy consultations or surgical schedules in our hospital. The significant reduction in NST physician response times following our intervention confirmed the necessity of enhanced NST−HIT for timely nutritional support.

**Figure 6:**
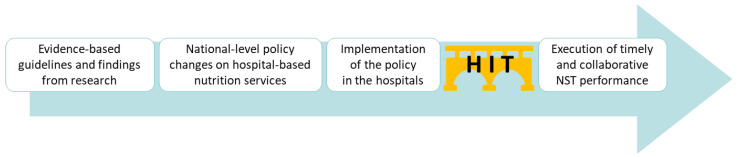
For timely and collaborative performance by the nutrition support team (NST), the role of healthcare information technology (HIT) is depicted in bridging the gap between policy implementation and the provision of clinical services at a hospital in South Korea.

Evidence-based and patient-centered care have been key elements of high quality healthcare services since the 1990’s [28,29]. The utilization of clinical practice tools, such as the European Society for Clinical Nutrition and Metabolism guidelines, was fundamental to improving the consistency of nutrition care and cost-effectiveness [30]. A comprehensive plan was needed to achieve lasting improvements in clinical practice [31]. In our study from a real-world healthcare setting, a well-structured and timely implementation of enhanced NST−HIT with top-down hospital-wide supports and bottom-up collaboration was needed to sufficiently maximize the efficiency of nutrition care. As presented in Figure 6, we believe that HIT can promise a number of solutions, as suggested in the literature [32,33]. In the process of the healthcare provision, HIT as a form of electronic health records, clinical decision support systems, and shared decision processes can facilitate the practical translation of policy to efficient clinical application with potential long-term implications in enhanced public health.

This study had some limitations. Firstly, this pre-test and post-test study had inherent limitations of non-randomized, uncontrolled study designs. Secondly, although we were able to report the reduced length of hospital stay as a clinical outcome of the intervention, other clinical outcomes such as morbidity, mortality, or hospital readmissions and patient-level humanistic outcomes could not be evaluated. Future studies on those outcomes are needed to demonstrate the value of the team-based NST intervention and the use of HIT on nutrition care process. Thirdly, we only evaluated the effect of HIT on nutrition support in SNUBH, but HIT varies globally; hence, our results are not representative of all hospital settings.

## 5. Conclusions

Our findings highlight the impact of enhanced nutrition support practice and NST−HIT (establishment of an integrated NST patient management program, improvement of response steps for NST attending physicians, and development of CDW) on NST performance. The enhanced nutrition support practice and NST−HIT significantly increased the timely completion rate of NST consultations and reimbursement rates, and reduced the length of hospital stay associated with the service by multidisciplinary NST. We believe that this study provides evidence of improvements in NST performance due to well-structured collaborative interventions implemented with the support of the hospital leadership. Our approach can be potentially applicable in other hospital settings and helpful for bridging gaps in supporting policies and improvement of public health.

## Figures and Tables

**Table 1:** Changes in administrative outcomes between pre- and post-intervention periods.

Outcomes	Pre-Intervention (1 January 2015−31 December 2015) Mean (SD)	Post-Intervention (1 July 2016−31 December 2018) Mean (SD)	*p*-Value	Mean Difference
Timely completion rate of NST consultation (%)	45.00 (8.24)	82.36 (4.68)	<0.001	37.36
Response time by professionals (hours)				
NST physicians	71.77 (29.98)	19.76 (4.64)	<0.001	−52.01
NST nurses	35.32 (10.29)	14.49 (6.20)	<0.001	−20.83
NST pharmacists	13.30 (1.87)	13.03 (1.34)	0.604	−0.27
NST dietitian	8.88 (1.13)	8.39 (0.90)	0.150	−0.49
Reimbursement rate (%)	51.36 (9.29)	78.65 (4.17)	<0.001	27.29

NST, nutrition support team; SD, standard deviation.

## Data Availability

Data sharing is not applicable to this article.

## Appendix A

**Table A1:** Nutrition screening index in SNUBH.

NSI = Age × 1 + BMI × 1.5 + Serum Albumin × 2 + TLC × 1.5
Age (years) > 65; 1, ≤65; 2 BMI (kg/m^2^) < 18.5; 1, ≥18.5; 2 Serum albumin (g/dl) < 3.5; 1, ≥3.5; 2 TLC (cells/mm^3^) < 900; 1, ≥900; 2

SNUBH, Seoul National University Bundang Hospital; NSI, nutritional screening index; BMI, body mass index; TLC, total lymphocyte count.

**Table A2:** Student’s *t*-test results of the time (hours) required to order TPN by ward physicians for patients with NST referrals during the pre- and post-intervention periods.

Group	*n*	Mean (SD)	*t*	*p*-Value
Pre-intervention ^1^	1262	22.81 (21.80)	4.421	<0.001
Post-intervention ^2^	1389	18.19 (20.38)		

NST, nutrition support team; SD, standard deviation; TPN, total parenteral nutrition. ^1^ January 2015–December 2015. ^2^ January 2017–December 2017.

**Table A3:** Student’s *t*-test results of the length of hospital stay (days) in non-critically ill adult inpatients receiving NST services during the pre- and post-intervention periods.

Group	*n*	Mean (SD)	*t*	*p*-Value
Pre-intervention ^1^	1171	32.87 (28.55)	2.358	<0.05
Post-intervention ^2^	2171	30.42 (28.61)		

NST, nutrition support team; SD, standard deviation. ^1^ January 2015–December 2015. ^2^ January 2018–December 2018.
